# Electrochemical Immunosensor for Detection of Aflatoxin B_1_ Based on Indirect Competitive ELISA

**DOI:** 10.3390/toxins10050196

**Published:** 2018-05-11

**Authors:** Farah Asilah Azri, Rashidah Sukor, Jinap Selamat, Fatimah Abu Bakar, Nor Azah Yusof, Reza Hajian

**Affiliations:** 1 Laboratory of Food Safety and Food Integrity, Institute of Tropical Agriculture and Food Security, Universiti Putra Malaysia, 43400 Serdang, Selangor, Malaysia; farah.asilah90@gmail.com (F.A.A.); sjinap@gmail.com (J.S.); 2 Department of Food Science, Faculty of Food Science and Technology, Universiti Putra Malaysia, 43400 Serdang, Selangor, Malaysia; ramlefatim@gmail.com; 3 Department of Chemistry, Faculty of Science, Universiti Putra Malaysia, 43400 Serdang, Selangor, Malaysia; azah1973@gmail.com; 4 Functional Devices Laboratory, Institute of Advanced Technology, Universiti Putra Malaysia, 43400 Serdang Selangor, Malaysia; rezahajian2002@yahoo.co.uk

**Keywords:** indirect competitive ELISA, electrochemical immunosensor, aflatoxin B_1_, multi-walled carbon nanotubes, chitosan, screen-printed carbon electrode, peanut

## Abstract

Mycotoxins are the secondary toxic metabolites produced naturally by fungi. Analysis of mycotoxins is essential to minimize the consumption of contaminated food and feed. In this present work, an ultrasensitive electrochemical immunosensor for the detection of aflatoxin B_1_ (AFB_1_) was successfully developed based on an indirect competitive enzyme-linked immunosorbent assay (ELISA). Various parameters of ELISA, including antigen–antibody concentration, blocking agents, incubation time, temperature and pH of reagents, were first optimized in a 96-well microtiter plate to study the antigen–antibody interaction and optimize the optimum parameters of the assay. The optimized assay was transferred onto the multi-walled carbon nanotubes/chitosan/screen-printed carbon electrode (MWCNTs/CS/SPCE) by covalent attachment with the aid of 1-Ethyl-3-(3-dimetylaminopropyl)-carbodiimide (EDC) and *N*-hydroxysuccinimide (NHS). Competition occurred between aflatoxin B_1_-bovine serum albumin (AFB_1_–BSA) and free AFB_1_ (in peanut sample and standard) for the binding site of a fixed amount of anti-AFB_1_ antibody. Differential pulse voltammetry (DPV) analysis was used for the detection based on the reduction peak of TMB_(ox)_. The developed immunosensor showed a linear range of 0.0001 to 10 ng/mL with detection limit of 0.3 pg/mL. AFB_1_ analysis in spiked peanut samples resulted in recoveries between 80% and 127%. The precision of the developed immunosensor was evaluated by RSD values (*n* = 5) as 4.78% and 2.71% for reproducibility and repeatability, respectively.

## 1. Introduction

Mycotoxins are toxic fungal metabolites that can contaminate primary food products as a result of mold growth. A large number of mycotoxins have been reported in peanuts, cereals (e.g., maize, sorghum, rice, wheat, barley and oats) and spices (e.g., black pepper, ginger, nutmeg, chili and turmeric). Moreover, some mycotoxins have been proven to be strong carcinogenic agents that are potentially hazardous to human and animal health [1]. Mycotoxins may be present in the food chain due to the ingestion of infected food by humans or as in infected ingredients in livestock feed, which can eventually lead to ‘mycotoxicoses’ (poisoning caused by mycotoxin) [2]. Major groups of mycotoxin include aflatoxins, ochratoxin A, fumonisins, deoxynivalenol, T-2 toxin and zearalenone [3]. Of these, aflatoxins are the most common and widely produced mycotoxin on food crops during their storage or preparation because of the favorable environmental conditions, especially temperature and humidity that are optimal for their growth [4,5]. Due to the high mycotoxin contamination in tropical countries, consumers are subjected to various health hazards [6]. Of all the 18 known analogues of aflatoxins, aflatoxin B_1_ (AFB_1_) is the most toxic, mutagenic, teratogenic and carcinogenic and its toxicity is ten times than that of potassium cyanide, 68 times of arsenic, 416 times of melamine, 70 times of dimethylnitrosamine and 10,000 times of benzene hexachloride [7]. It is also classified as a group 1 carcinogen by the International Agency for Research on Cancer (IARC) [8].

Due to the significant health risks related with the occurrence of aflatoxins in food, and also to gratify the severe legal requirement, it is vital to have efficient techniques for the detection [9]. Enzyme-linked immunosorbent assay (ELISA) is one of the alternative methods for detection of aflatoxins. It is an immunoassay that works based on selective and sensitive antibody–antigen (Ab–Ag) interaction. ELISA has several advantages such as simple, sensitive, low cost and the use of safe reagents [10]. Currently, most commercially available ELISA kits for the detection of mycotoxins are functioning in the kinetics phase of antibody–antigen binding, which eventually can shorten the incubation time [11]. Competitive binding of an antibody is more suitable for low molecular weight compounds such as mycotoxins since it has single antigenic determinant. According to Orlov et al. [12], limited reproducibility in (i) surface functionalization and (ii) preparation of “carrier protein—hapten” conjugates to be immobilized on the surface; this is one of the most challenging concerns for competitive immunoassays, which can cause significant deviations in the quantity of antibody binding sites. The concept of these antibody–antigen interactions can be applied in the development of the biosensor. A biosensor is a device that can determine the analyte based on the incorporation of bioactive materials with physiochemical transducing element [13]. The bioactive material can be classified as affinity or biocatalytic such as antibodies, DNA, receptor protein, enzymes, tissues, whole cells or organs interrelating with specific analyte. The interaction can then be converted by the transducer into a quantifiable electrical signal.

An antibody-based biosensor is also known as immunosensor. For the past years, many sensitive electrochemical immunosensors have been developed for the detection of AFB_1_ with the range of detection reported to be as low as 0.03–0.15 µg/L [14,15,16,17,18,19,20,21,22,23,24,25]. At the same time, various types of nanomaterial have been explored to be incorporated in the sensor development due to their unique physicochemical properties, i.e., increased sensitivity and enhanced current production [26]. A study done by Linting et al. [27] described an immunosensor for AFB_1_ with an enhanced electrochemical performance based on graphene/conducting polymer/gold nanoparticles/the ionic liquid composite film. The use of nanomaterials for electrode modification can remarkably improve electron transfer rate and can expand the electrochemical stability of the sensor in order to avoid losing the antibody during analysis. Meanwhile, having the same format as we proposed in this present work, Zhang et al. [25] developed an electrochemical immunosensor for detection of AFB_1_ in corn using single-walled carbon nanotubes/chitosan based on an indirect competitive binding which can quantitatively detect AFB_1_ from 0.01 to 100 ng/mL with the detection limit of 3.5 pg/mL. However, detailed study on the optimization of ELISA was not described. 

Herein, the aim of the present work was therefore to develop a sensitive electrochemical immunosensor for the detection of AFB_1_ in food samples, i.e., peanuts, based on indirect competitive ELISA. Several parameters which influenced the performance of ELISA, including type of blocking agent, concentration of coating conjugate, concentration of primary antibody, pH of buffer, incubation temperature and incubation time, were first optimized using multi-level factorial design. Factorial design was used to study the effects of the factors towards the response when interactions may be present but the levels of each factor are not similar [28]. This optimization is important to maximize the response of the specific binding of antibody–antigen and produce higher signal to noise ratio before being applied in an electrochemical system. The optimized ELISA was then transferred onto the screen-printed carbon electrode (SPCE) that was modified with multi-walled carbon nanotubes (MWCNTs) and chitosan (CS), which was proven in our previous work to significantly increase the sensitivity and conductivity [29]. Previously, we had successfully modified the surface of the SPCE with the functionalized MWCNTs by introducing −COOH onto the surface of the MWCNTs. Few parameters have been optimized to maximize the performance of the working electrode, including ratio of MWCNTs:CS, coating volume and drying condition. This modification greatly enhanced the electron transfer rate and increased the electrode active surface area for further immobilization of bioactive components, which will be further discussed in the present work.

## 2. Results and Discussion

### 2.1. Optimization of the ELISA Parameters

AFB_1_ is a hapten which has only one epitopic site on its molecule. Hence, a competitive indirect immunoassay was adapted in the present work for ELISA optimization. The binding specificity of the primary antibody (rabbit anti-AFB_1_) to the binding site of the coating conjugate (AFB_1_–BSA) was examined through the checkerboard titration method. The concentrations of AFB_1_ standards immobilized on the microtiter well were varied from 0.00001 to 1000 ng/mL, just like the concentrations of rabbit anti-AFB_1_ antibody which were in the dilution range of 1/2500 to 1/640,000 (*v*/*v*). The concentration of the secondary antibody (goat anti-rabbit horseradish peroxidase (HRP) conjugate) was kept constant at 1/5000 (*v*/*v*) dilution. Based on the result, decreasing the concentrations of both coating conjugate and antibody might cause the assay to have higher sensitivity. The absorbance decreased as lower concentrations of coating conjugate and antibody were used, although the signal plateaued at 1/10,000 (*v*/*v*) of rabbit anti-AFB_1_ antibody. Decreasing the concentrations of both coating antigen and antibody caused the coated antibody to bind with lower analyte which in turn caused the antibody to better recognize the analyte and subsequently increase its sensitivity. 

A few combinations of coating conjugate and antibody concentrations that showed favorable signal were subjected to further assay using indirect competitive format. This is because the sensitivity of the ELISA is highly dependent on the antibody recognition towards free antigen as compared to the bound antigen. Meanwhile, the concentrations of coating conjugate and antibody should be sufficiently high to produce a measurable signal for antigen–antibody binding, and they should also be sufficiently low to maximize the competition with free analyte. The effect of AFB_1_–BSA concentrations of the coating conjugate and anti-AFB_1_ from rabbit as the primary antibody towards the indirect competitive ELISA with their best-fit values are shown in Table 1. The half maximal inhibitory concentration (IC_50_) is a measure of the effectiveness of a substance in inhibiting a specific biological or biochemical function. This quantitative measurement indicates how much a particular substance (inhibitor) is needed to inhibit a given biological process. Therefore, IC_50_ values were also evaluated in the present work. 

Based on the result, 0.25 µg/mL of coating conjugate with 1/5000 (*v*/*v*) of anti-AFB_1_ antibody (dilution B) had the lowest IC_50_ values of 0.018 ng/mL and the highest top value on *y*-axis (A)/bottom value on *y*-axis (D), A/D value of 73.0. Moreover, the IC_50_ values of dilutions A and B were almost the same from each other (i.e., 0.024 ng/mL and 0.018 ng/mL, respectively). However, dilution A had a lower A/D value, i.e., 32.73 as compared to dilution B, i.e., 73.00. Meanwhile, dilutions C, D and E, which were coated with 1.0 µg/mL of AFB_1_–BSA were introduced with different concentrations of primary antibody. The results showed an unfavorable response particularly for dilution C (IC_50_ = 2.457 ng/mL). This finding indicates that higher concentrations of coating conjugate and antibody reduced the sensitivity of the assay, which subsequently increased the binding of antibody onto the coating conjugate rather than the free antigen. Based on the result, the IC_50_ value for dilution F was significantly low, i.e., 0.004 ng/mL, which could be due to an excessive amount of anti-AFB_1_ antibody, with limited binding site to the coating conjugate. Therefore, less competition occurred between the free antigens and coating conjugate. Decreasing both the concentrations of dilution B allowed the antibody to bind with lower analyte on the plate and enhanced the sensitivity. Thus, this combination (0.25 µg/mL of AFB_1_–BSA; 1/5000 *v*/*v* of anti-AFB_1_) was chosen as the optimal concentration for optimization of the assay.

**Figure 1:**
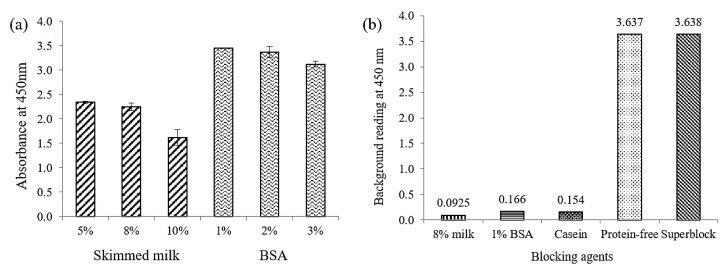
(**a**) Absorbance reading of different blocking agents (skimmed milk and BSA) at various concentrations in non-competitive ELISA. Error bars indicate = SD, *n* = 3. (**b**) Effect of various blocking agents on background reading by eight percent skimmed milk, one percent BSA, casein, protein-free and superblock.

The optimization of the assay was furthered by determining the optimal blocking agent. Blocking agent is crucial in ELISA system to reduce the non-specific binding of proteins as well as to produce low background readings. A variety of blocking buffers including synthetic and protein polymers have been studied based on their ability to block unreacted binding sites on the solid surface [30]. Five different types of blocking agent were chosen in the present work to investigate the blocking reaction on the surface of the wells, which included skimmed milk, bovine serum albumin (BSA), casein, protein-free and superblock. The concentration of skimmed milk and BSA were first optimized before being compared with other type of blocking agents. Figure 1a shows that five percent and eight percent skimmed milk gave higher absorbance reading compared to 10% skimmed milk. However, eight percent skimmed milk resulted in lower background reading in general. Similar to one percent and two percent BSA which were not different from each other in terms of the absorbance readings at 450 nm, but one percent BSA produced a lower background reading. Therefore, eight percent and one percent were chosen as the optimal concentration for skimmed milk and BSA, respectively. Meanwhile, Figure 1b shows the background reading obtained for each of the blocking agents. It is apparent that protein-free and superblock produced the highest background reading as compared to eight percent skimmed milk, one percent BSA and casein. High background reading might be caused by the stickiness of the antibodies attached to the surface of the microtiter wells [31]. Skimmed milk (eight percent) diluent showed the lowest background reading followed by casein and one percent BSA. This indicates that the most efficient blocker in this assay was skimmed milk as it was able to reduce non-specific binding and subsequently enhanced the signal/background (S/B) ratio to produce a sensitive assay. 

**Figure 2:**
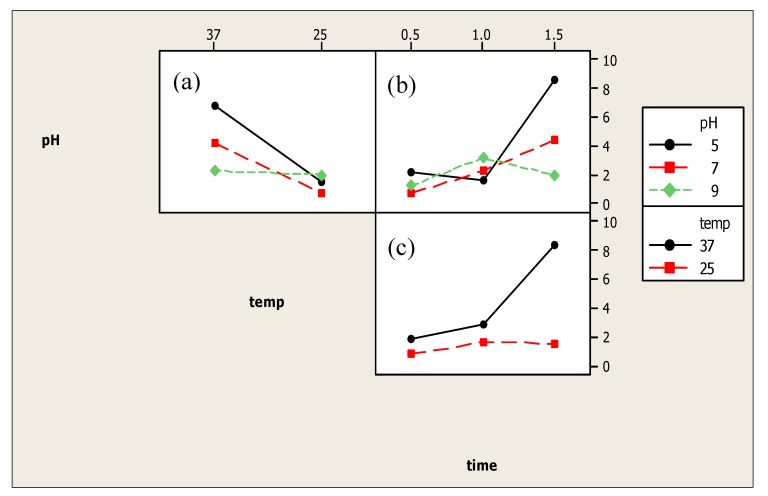
Interaction plot between (**a**) pH of buffer and incubation temperature, (**b**) incubation time and pH of buffer (**c**) incubation time and incubation temperature, based on the inhibitory concentration (IC_50_) value obtained from the absorbance reading of the indirect competitive ELISA.

Incubation time, incubation temperature as well as pH of buffer contributed significant effects on the performance of the assay. Figure 2 shows the interaction plot of the three levels (incubation time, incubation temperature and pH of buffer) against the IC_50_ value. Result of the analysis of variance for the IC_50_ values indicates no significant difference for the factors of pH and incubation time, with *p* value of 0.328 and 0.148, respectively. However, incubation time contributed to a significant effect (*p* < 0.05) towards the IC_50_ of the assay, with *p* value of 0.036. Figure 2a shows the interaction between incubation temperature and pH. Based on the result, IC_50_ values for all the three pH values were lower at 25 °C as compared to 37 °C. Regardless of the incubation time, pH 7.0 at incubation temperature of 25 °C produced the lowest IC_50_ value for this interaction. Meanwhile, Figure 2b displays the interaction between pH and incubation time, which resulted in a dual-effect response. Specifically, the IC_50_ value of pH 5.0 was found to decrease when the incubation time was increased to 1.0 h. However, the IC_50_ value increased when the incubation time was prolonged to 1.5 h. Similarly, at pH 9.0, the plot shows that the IC_50_ value at 1.0 h of incubation time was the highest as compared to 0.5 and 1.5 h. pH 7.0 gave a better result as the IC_50_ value increased when the incubation time was increased. 

Regardless of the incubation temperature, pH 7.0 at 0.5 h incubation temperature gave the lowest IC_50_ value for this interaction. Figure 2c shows the interaction plot between incubation time and incubation temperature. Based on the result, both temperatures (25 °C and 37 °C) gave a dual-effect response. Nonetheless, the IC_50_ values of incubation temperature at 25 °C were not significantly different (*p* > 0.05) for all the three incubation times (0.5 h, 1.0 h and 1.5 h) as compared to the incubation at 37 °C. Therefore, regardless of the buffer’s pH of the assay incubated at 25 °C, 0.5 h gave a better response based on the lowest IC_50_ value yielded. Based on all the interaction plots discussed, it can be concluded that this ELISA has performed well in the buffer at pH 7.0, with 0.5 h of incubation time at 25 °C. Thus, these optimal conditions were used for further work in developing the immunosensor.

### 2.2. Standard Calibration Curve of AFB_1_ in Spectrophotometric ELISA

**Figure 3:**
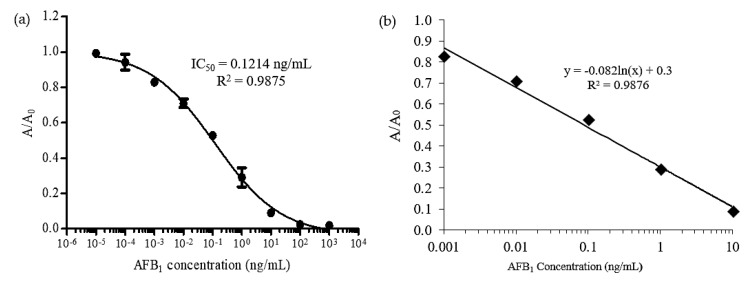
(**a**) Calibration curve of AFB_1_ for indirect ELISA using spectrophotometric detection. Wells were coated with AFB_1_–BSA (0.25 µg/mL), blocked with 8% skim milk and followed by competition between anti-AFB_1_ (1/5000, *v*/*v*) and free AFB_1_ (0–1000 ng/mL) before adding the anti-rabbit IgG horseradish peroxidase (HRP) (1/5000, *v*/*v*). Error bar = standard deviation, *n* = 3. (**b**) Linear regression of standard curve with AFB_1_ working range from 0.001 to 10 ng/mL. The curve was fitted by non-linear regression using the four-parameter logistic equation.

The data collected from the previous ELISA experiment using various concentration of AFB_1_ was interrogated into a standard curve plot before the quantity of analyte can be measured and quantified. The competitive standard curve was inversely proportional between the signals and the analyte concentrations. The response of the signal was relative to the amount of enzyme conjugate (HRP) bound to the assay, based on the competition of free AFB_1_ and AFB_1_–BSA to the antibody. Based on Figure 3a, a non-linear calibration curve was obtained. The A/A_o_ reading decreased with the increase of concentration of AFB_1_ standard solution. Based on the result, higher signals were observed from 0.0001 to 0.001 ng/mL, then dramatically decreased after 100 ng/mL. High signals indicated that high amount of primary antibody was bound to the antigen coated in the wells, which means no or less free analyte (AFB_1_ standard) was bound to the antibody. From the result, the linear range of AFB_1_ detection was between 0.001 to 10 ng/mL of with R^2^ value of 0.9875. Figure 3b shows the linear plot within the working range to obtain the equation for this assay. The limit of detection (LOD) was calculated as mean ± 3SD. The value was inserted into the GraphPad Prism software and the interpolated x-value was obtained. The obtained LOD for spectrophotometric ELISA was 0.0015 ng/mL.

### 2.3. Study of the Enzyme–Substrate Interaction

Having the same format as in spectrophotometric ELISA, the horseradish peroxidase (HRP) activity is an important factor that needs to be studied. After the assay has been successfully transferred onto the surface of SPCE, the system could then be incorporated into an electrochemical format. The HRP enzyme catalyzed the mediator (TMB) to be reacting with hydrogen peroxide (H_2_O_2_). Therefore, the electroactivity of TMB/H_2_O_2_ in a phosphate-buffered saline (PBS, pH 7.0) was first investigated by Cyclic Voltammetry (CV) to determine the redox profile. Based on the results, MWCNTs/CS/SPCE immobilized ELISA in blank PBS had no oxidation and/or reduction peak, while CV in the presence of H_2_O_2_ and TMB mediator in PBS had one oxidation peak at the potential of +0.3 V and one reduction peak at +0.1 V (data not shown). Therefore, the electrochemical behavior of this substrate was involved in the situation where TMB_(red)_ was oxidized at the oxidation peak of +0.3 V, while its product TMB_(ox)_ was reduced at +0.1 V. Heurich and Kadir also found a similar behavior of TMB_(red)_ and TMB_(ox)_ on glassy carbon electrode and screen-printed gold electrode, respectively [32,33]. In the presence of HRP enzyme as a catalyst upon addition of secondary antibody with peroxidase conjugate (goat anti rabbit IgG-HRP) to the TMB/H_2_O_2_ substrate solution, only one reduction peak remained at the potential of +0.1 V. This result could suggest that when the secondary antibody was added, the mediator TMB was completely and oxidized to TMB_(ox)_ [31]. The HRP activity can be determined based on the reduction peak produced by TMB_(ox)_. The peak current of TMB_(ox)_ is proportional to the HRP enzyme because the electrochemical product (TMB_(ox)_) is regenerated by the enzyme [34].

### 2.4. Chronoamperometry Study of Enzyme Activity Using TMB/H_2_O_2_

Chronoamperometry was used to characterize the change in current with the addition of TMB substrate and H_2_O_2_ for the activity of HRP enzyme. This analysis implicates stepping the potential of the working electrode from the values at which no faradic reaction occurs to a steady state potential at which the surface concentration of the electroactive species is effectively zero [35]. At a constant potential of −0.1 V, the potential for HRP activity can be determined at the reduction current generated by TMB_(ox)_. Based on the result, there was an obvious change observed in the current response. The current increased even further as a result of the reduction of H_2_O_2_ catalyzed by HRP_(red)_ and the oxidized product, where HRP_(Ox)_ could chemically react with TMB_(red)_ and convert to its initial form, TMB_(Ox)_. This proves a high enzyme response towards the catalytic reaction. HRP had a direct enhancement on the response in which the generated current was proportional to the amount of antibody HRP conjugate bound to immunoassay on the electrode surface. However, this current was indirectly proportional to the concentration of targeted antigen being tested.

### 2.5. Analytical Performance of Designed Immunosensor

Based on the CV studies, a reduction peak was observed at the potential of ~+0.1 V. Therefore, a DPV analysis was performed in the potential range of +0.1 to +0.4 V for the quantitative analysis of AFB_1_ using PBS containing 0.15M NaCl as the supporting electrolyte. The cathodic peak resulted from the reduction of TMB_(ox)_ was clearly shown at potential between +0.2 to +0.3 V. Based on the result, the current was increased after the addition of HRP enzyme, indicating that H_2_O_2_ had been reduced, as shown in this reaction. This subsequently increased the reduction of TMB_(ox)_ at the surface of electrode. This result confirms that the peak potential of 0.25 V ± 0.1 is useful for the detection of AFB_1_. 

**Figure 4:**
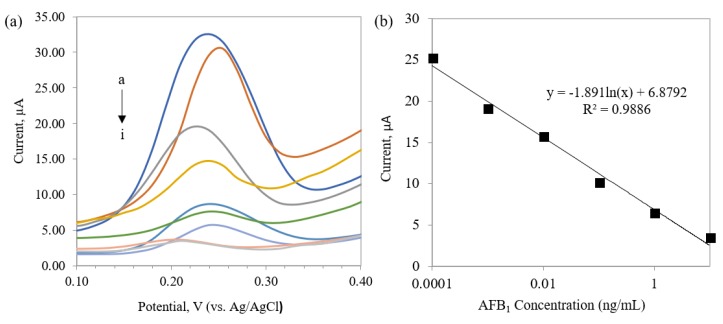
(**a**) Differential pulse voltammetry (DPV) peak currents of different concentrations of AFB_1_ (a) blank (b) 0.0001 ng/mL (c) 0.001 ng/mL (d) 0.01 ng/mL (e) 0.1 ng/mL (f) 1 ng/mL (g) 10 ng/mL (h) 100 ng/mL (i) 1000 ng/mL, within potential range of 0.1 to 0.4 V. (**b**) The regression of peak currents vs. different AFB_1_ concentrations. The curve was fitted by non-linear regression using the four-parameter logistic equation.

**Figure 5:**
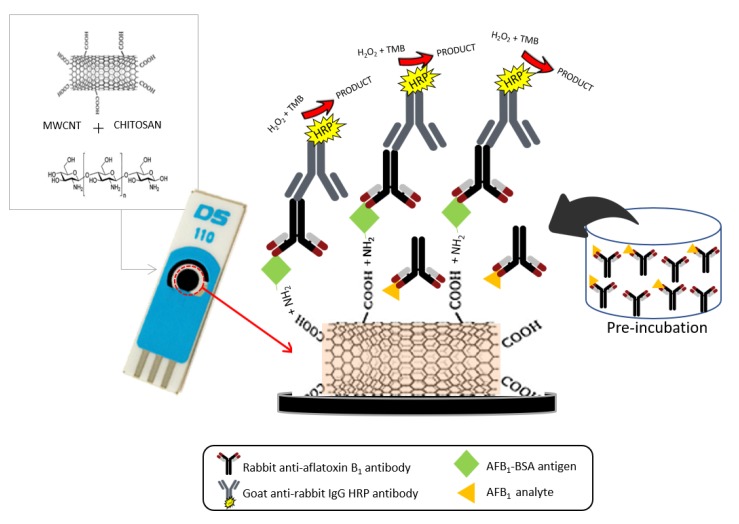
The complete schematic diagram of the nanomaterial-based immunosensor based on ELISA indirect competitive format. The primary antibody (rabbit anti-AFB_1_ antibody) was first pre-incubated with AFB_1_ prior to transferring onto the electrode surface. The remaining antibodies will bind to the antigen (AFB_1_–BSA) which were immobilized on the surface while the pre-occupied antibodies will be washed away during the washing step.

In order to evaluate the performance of the electrochemical immunosensor, different concentrations of AFB_1_ were assayed. Figure 4a shows the current response based on the enzyme activity of HRP at different concentrations of AFB_1_. As it has shown, there is an inverse correlation between immunosensor response and AFB_1_ concentration because there is affinity between AFB_1_ and primary antibody in the solution (Figure 5). Therefore, during the washing step, it would be eliminated from the assay. As a result, less or none of the primary antibody was available to bind to the antigen (AFB_1_–BSA) which was immobilized on the electrode surface. Figure 4b presents the plot of calibration curve for AFB_1_ using the DPV peak current. Data of the plots are illustrated by a linear regression in a working range between 0.0001–10 ng/mL (R^2^ = 0.9886). The limit of detection (LOD) was 0.3 pg/mL, which was calculated based on mean ± 3SD. The results found from the present work are comparable with our previous work in feed sample, in which the working range was from 0.0001 to 10 ng/mL with LOD of 0.1 pg/mL [36]. 

The precision of the immunosensor system was evaluated by calculating the percentage of relative standard deviation (%RSD) value. Reproducibility of the developed immunosensor was performed by fabricating five SPCEs separately and evaluating them based on indirect competitive ELISA in supporting electrolyte by DPV analysis. Meanwhile, the repeatability of the immunosensor was identified by scanning the same fabricated SPCE for the same sample five times. From the results, the %RSD of the developed immunosensor was 4.78% and 2.71% for reproducibility and repeatability, respectively. However, electrochemical immunosensors suffer from insufficient stability over extended period of time [37]. In this study, the use of EDC–NHS and nanocomposite of MWCNT/CS had a positive effect on the immobilization of the antibody on the active site of sensor. 

### 2.6. Detection of AFB_1_ in Peanut Samples

The performance of the developed immunosensor was then examined in real food samples. Peanut is a common commodity that contains a high amount of AFB_1_. Blank peanut sample was spiked with respective AFB_1_ concentrations. The extract of peanut sample, especially in solid based samples, can contribute to a matrix effect. The detection efficiency of mycotoxins in food matrix highly depends on the efficiency of the sample preparation. Extraction solvents selected usually depend on the physical and chemical characteristics of the sample, the solvent cost and safety, and the solubility of the non-analyte in the extraction solvent [38]. The use of water in extraction procedure usually increases the extraction efficiency because water can break the interactions between toxins and other sample constituents and subsequently improves the penetration of the solvent into the material [39]. Based on the results in Table 2, good recovery values between 80–127% (*n* = 3) were observed in both the detection methods. The extraction method used was without the sample clean-up or pre-treatment as in the sample preparation for HPLC analysis. The present work demonstrated that the matrix effect from the peanut sample was minimal. The pre-treated method showed a greater recovery due to the removal of interference of co-extracted compounds in the sample. However, the recovery result was still reliable for the detection of AFB_1_ in the sample matrix. Therefore, one of the advantages of the developed immunosensor is that the necessity of a sample pre-treatment can be avoided.

## 3. Conclusions

The present work demonstrates and reports an ultrasensitive electrochemical immunosensor for the detection of AFB_1_ using modified SPCE/MWCNTs/CS. The optimum ELISA system was successfully transferred to the SPCE by activating the carboxylic acid groups at the surface of MWCNTs and forming a stable amide bond with AFB_1_–BSA. For the electrochemical detection, signal was detected by using the electron produced from the reduction of TMB_(ox)_ to TMB_(red)_ with the aid of HRP enzyme at a constant potential between +0.2 and +0.3 V. Furthermore, the developed electrochemical immunosensor has shown a linear working range of 0.0001 to 10 µg/L with the limit of detection of 0.3 pg/mL. In addition, this immunosensor can be applied in real peanut samples without extensive sample preparation. The test carried on the spiked sample showed an acceptable recovery percentage of 80–127%. As a conclusion, the developed immunosensor can be a great tool for food safety and quality monitoring where presence of mycotoxins is concerned.

## 4. Materials and Methods

### 4.1. Reagents

AFB_1_–BSA, rabbit anti-AFB_1_, goat anti-rabbit IgG horseradish peroxidase (HRP) conjugate, 3,3′,5,5′-tetramethylbenzidine (TMB) substrate, carbonate–bicarbonate buffer (capsule), multi-walled carbon nanotubes (MWCNTs), medium molecular weight chitosan (CS) and potassium ferricyanide, K_3_[Fe(CN)_6_], were purchased from Sigma-Aldrich Co. (St. Louis, MO, USA). Aflatoxin B_1_ (AFB_1_) standard solution was purchased from Supelco Analytical (Bellefonte, PA, USA). Skimmed milk powder was obtained from Nacalai Tesque (Kyoto, Japan). Other reagents were of analytical grade, and all aqueous solutions were prepared using deionized water. Phosphate buffer saline (PBS) was prepared at 10× stock concentration and diluted to 1×. Tween 20 (0.05%) was added to 1 L of 1× PBS to make PBST as a washing buffer in ELISA. For supporting electrolyte, 0.17 g of potassium ferricyanide, K_3_[Fe(CN)_6_], was added into 100 mL of 1× PBS to make a 5 mM solution, to study the reversible electrochemical behavior in cyclic voltammetry analysis.

### 4.2. Apparatus

All the electrochemical measurements including Cyclic Voltammetry (CV), Differential Pulse Voltammetry (DPV) and chronoamperometry were carried out by using Autolab Electrochemical Analyzer, µAUTOLAB Potentiostat/Galvanostat (µ3Aut71052) and the current was analyzed by General-Purpose Electrochemical Software (GPES, version 4.9, Autolab @ Eco Chemie, Utrecht, The Netherlands, 2004). All the measurements were recorded by using disposable screen-printed carbon electrodes (SPCE) from DropSens (Metrohm, Herisau, Switzerland). The SPCE is made up of ceramic (L33 × W10 × H0.5 mm) with silver electric contacts. It consists of three types of electrodes which are working (carbon, 4 mm diameter), counter (carbon) and reference (silver).

### 4.3. Optimization of ELISA

An indirect competitive ELISA was optimized through checkerboard ELISA method to obtain the optimal concentration of coating conjugate and primary antibody. The competitive ELISA was done by coating a 96-well microtiter plate with AFB_1_–BSA at 0.25 µg/mL which had been pre-determined from the checkerboard ELISA method. The plates were incubated at 4 °C overnight (16 h). The next day, the plates were blocked with 1% BSA in PBS for 1 h at room temperature with gentle shaking. AFB_1_ standards (50 µL), ranging from 10^−4^ to 10^3^ ng/mL in 10% methanol and 50 µL of rabbit anti-AFB_1_ (1/5000, *v*/*v*) were added simultaneously into the wells. AFB_1_ standard was allowed to compete with the coating conjugate (AFB_1_–BSA) for antibody binding side for 1 h at room temperature with continuous shaking. Then, goat anti-rabbit HRP conjugate was added at (1/5000, *v*/*v*) and the plates were further incubated for another hour at room temperature with gentle shaking. TMB substrate was added and a reaction was developed for 30 min in the dark at room temperature. Then, the reaction was stopped by adding 1 N H_2_SO_4_, and absorbance was read at 450 nm. Washing with PBST was required after each step. The conditions of assay, including pH of buffer, incubation time and incubation temperature were optimized using multi-level factorial design. 

### 4.4. Fabrication of the Electrode

SPCE was modified by using nanocomposite of MWCNTs/CS based on method described by Azri et al. [29]. The MWCNTs was functionalized by using H_2_S0_4_:HNO_3_ (3:1) treatment to add carboxylic acid groups on the surface to increase the surface area. Five milligrams of MWCNTs powder was dispersed into 1 mL of 0.5% chitosan solution (prepared in acetic acid). Then, the mixture was sonicated for 1 h so as to allow the carbon to mix well with the chitosan solution and produce uniform black suspension. Meanwhile, 10 µL of the dispersion was added to working electrode and dried at 80 °C in an oven for 10 min to obtain MWCNTs/CS/SPCE. 

### 4.5. Design of Electrochemical Immunosensor

Prior to transferring the optimized indirect competitive ELISA onto the MWCNTs/CS/SPCE, 20 µL of activated fluid containing EDC–NHS mixture (0.4 M EDC and 0.2 M NHS) was added to the electrode surface for 1 h at room temperature to activate carboxylic acid groups, without purification steps. By activation of −COOH groups on MWCNTs, two parallel reactions occurred: firstly, covalent binding between −NH_2_ of chitosan with activated −COOH and secondly, covalent binding between −NH_2_ of BSA with activated −COOH. Then, MWCNTs/CS/SPCE was washed thoroughly using PBS. Using similar steps in spectrophotometric ELISA, the format was transferred onto MWCNTs/CS/SPCE with a minor modification done to the added volume. Specifically, ten microliters of reagent were used instead of 100 µL (for 96-well microtiter plate). Ten microliters of AFB_1_–BSA was added to the modified SPE and incubated for another 2 h at room temperature or overnight at 4 °C. The electrode was then washed with PBS and blocked for an hour using 8% skimmed milk solution. Various concentrations of AFB_1_ standard (0.0001 to 1000 ng/mL) and anti-AFB_1_ antibody were pre-incubated at room temperature for 0.5 h before being added onto the electrode. The assay was then added with goat anti-rabbit IgG conjugated HRP (10 µL, 1:5000 *v*/*v*) in PBS and incubated for 1 h. The electrode was washed thoroughly three times within each step. The design of the immunosensor is illustrated in Figure 5. 

### 4.6. Electrochemical Reaction

**Figure 6:**
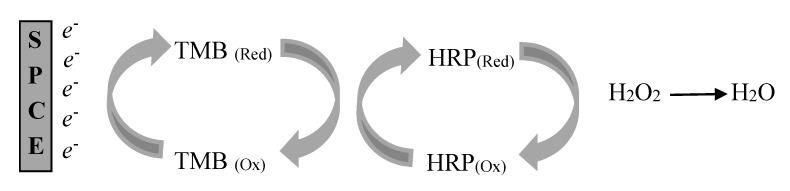
Schematic diagram of the catalytic chemical reaction of TMB on the surface of multi-walled carbon nanotubes/chitosan/screen-printed carbon electrode (MWCNTs/CS/SPCE) in the presence of HRP enzyme as catalyst.

All measurements were performed by adding 100 µL of TMB and 0.05% H_2_O_2_ in 0.1 M PBS containing 0.15 M NaCl. In the developed immunosensor, TMB was used as the electroactive mediator because it can be reduced directly on the surface of electrode. In this system, hydrogen peroxide in the solution was the first to be reduced by the immobilized HRP. Then, HRP_(Ox)_ was regenerated to HRP_(Red)_ with the aid of the mediator, TMB_(Red)_, via chemical oxidation to TMB_(Ox)_. Subsequently, the oxidized TMB was electrochemically reduced on the electrode surface and this increased the reduction current. The mechanism of the chemical reaction in the developed immunosensor is shown in Figure 6. The electrochemical response in DPV was optimized by manipulating the setting of step potential which influenced the scan rate. Electrochemical measurements were performed by using CV, Chronoamperometry and DPV analysis.

### 4.7. Sample Preparation

The unshelled peanuts were purchased from a local wet market in Sri Serdang, Selangor, Malaysia. Sample preparation and extraction were done based on the technique described by Ammida et al. [9] with minor modifications. One hundred grams of peanut was ground to powder using a blender. Five grams of the ground peanut was spiked with 500 µL AFB_1_ standard (0.1, 1, and 10 ng/mL). The samples were mixed using vortex for 1 min. Ten microliters of extraction solvent (85 methanol:15 PBS, *v*/*v*) was added and agitated in a shaker for 30 min at room temperature at 100 rpm. Then, the mixture was centrifuged at 6000 rpm for 10 min. The supernatant (1 mL) was collected and further diluted (1:1, *v*/*v*) with PBS for further analysis with ELISA to reduce the matrix effect.

## Figures and Tables

**Table 1:** Effect of concentrations of AFB_1_–BSA as coating conjugate and anti-AFB_1_ from rabbit as primary antibody towards the indirect competitive enzyme-linked immunosorbent assay (ELISA).

Coating Antigen Concentration, AFB_1_–BSA (µg/mL)		Primary Antibody Concentration, Anti-AFB_1_ (*v*/*v*)	Coefficient of Determination, R^2^	IC_50_ (ng/mL)	Hill Slope	Top Value on *y*-axis, A	Bottom Value on *y*-axis, D	A/D
A	0.5	1/10,000	0.979	0.024	−0.325	1.080	−0.033	32.73
B	0.25	1/5000	0.991	0.018	−0.420	1.022	−0.014	73.00
C	1.0	1/2500	0.995	2.457	−1.798	0.984	0.254	3.874
D	1.0	1/20,000	0.969	0.056	−0.650	0.973	0.106	9.179
E	1.0	1/10,000	0.913	0.059	−0.317	1.038	0.196	5.296
F	0.25	1/2500	0.967	0.004	−0.239	1.176	0.029	40.55

**Table 2:** Determination of AFB_1_ in spiked peanut sample by immunosensor and spectrophotometric ELISA (*n* = 3, mean ± SD).

Detection Method	AFB_1_ Concentration (ng/mL)		% Recovery
	Spiked	Detected ± SD	
Electrochemical Immunosensor	0	0.008 ± 0.005	-
	0.1	0.12 ± 0.028	111.8
	1	1.28 ± 0.33	127.1
	10	10.75 ± 0.67	107.5
Spectrophotometric ELISA	0	0.004 ± 0.002	-
	0.1	0.087 ± 0.015	83.2
	1	1.14 ± 0.28	113.6
	10	8.03 ± 0.91	80.3
